# Case Report: Progressive interstitial lung disease secondary to Sjögren’s disease in a patient with inclusion body myositis complicated by dysphagia—a multidisciplinary approach and therapeutic challenges

**DOI:** 10.3389/fmed.2025.1683606

**Published:** 2025-11-11

**Authors:** Emanuele Cocchiara, Francesca Cozzini, Ilaria Chiapparoli, Francesco Falco, Gianluigi Bajocchi, Carlo Salvarani, Andreina Manfredi

**Affiliations:** 1 Rheumatology Unit, IRCCS Santa Maria Novella Hospital, Reggio Emilia, Italy; 2 University of Modena and Reggio Emilia, Modena, Italy; 3 Pneumology Unit, IRCCS Santa Maria Novella Hospital, Reggio Emilia, Italy

**Keywords:** inclusion body myositis, Sjögren’s disease, interstitial lung disease, nintedanib, dysphagia

## Abstract

We present the case of a 78-year-old man with a complex overlap of inclusion body myositis (IBM) and Sjögren’s disease (SjD), complicated by interstitial lung disease (ILD) and esophageal dysfunction. The patient’s neuromuscular decline was managed with periodic intravenous immunoglobulins (IVIG), while his progressive ILD required a combined immunosuppressive and antifibrotic regimen, including prednisone. This case underscores the diagnostic and therapeutic challenges in managing autoimmune overlap syndromes, particularly the rare coexistence of IBM and SjD-related ILD. We highlight the rationale behind the treatment strategy and the importance of a tailored, multidisciplinary approach in addressing both muscular and pulmonary manifestations.

## Introduction

Inclusion body myositis (IBM) is an idiopathic inflammatory myopathy with insidious onset, often presenting with progressive muscle weakness and dysphagia (1).

It is characterized by slowly progressive muscle weakness, frequently accompanied by dysphagia and poor response to immunosuppressive therapies (1).

Although rare, its association with connective tissue diseases such as Sjögren’s disease (SjD) has been reported in the literature (2, 3).

The overlap between IBM and Sjögren’s syndrome (SS) can complicate diagnosis and management, particularly when interstitial lung disease (ILD)—a known extraglandular manifestation of SjD—coexists.

This case report describes a challenging patient with IBM, SjD, and SjD-related ILD. Given the rarity of this specific overlap and the need for a coordinated, multidisciplinary treatment approach, we aimed to shed light on the clinical decision-making process, the rationale for the chosen therapeutic strategy, and the broader implications for managing autoimmune overlaps with pulmonary involvement.

### Patient information

We present the case of a 78-year-old man, who was a former heavy smoker (50 cigarettes/day until 1990), with several comorbidities, including hypertension, cardiopathy, hypothyroidism, benign prostatic hyperplasia, osteoporotic L1 fracture, right ankle arthrodesis, suspected transient ischemic attack (TIA), occult hepatitis B virus (HBV) infection, and prior surgery for intestinal obstruction. The patient presented for the first time to the rheumatologic evaluation because of the occurrence of a clinical picture characterized by progressive dysphagia, muscle weakness, and dyspnea.

### Clinical findings

Careful diagnostic workup was performed to investigate the diagnosis. In 2017 at the age of 70 years, a diagnosis of IBM was confirmed after a muscle biopsy. At disease onset, the clinical picture was characterized by severely impaired muscle strength; on physical examination, the patient showed a strength deficit of finger flexors, wrist extensors, and knee extensors. He also presented esophageal dysmotility, which was confirmed by double-contrast radiography (RX). The examination showed an altered passage of liquid barium contrast in the patient’s esophagus, indicating a muscular disorder. Examinations revealed a creati kinase of 984 U/L and an aldolase of 20.5 U/L, while myositis antibodies were negative. Electromyography showed signs of acute multiradicular denervation, and a magnetic resonance (MR) imaging also revealed muscle edema and fatty degeneration of the left vastus lateralis muscle and bilateral gastrocnemius muscles. The muscle biopsy revealed endomysial lymphocytic (CD8-positive T lymphocyte) inflammation, atrophic fibers, and multiple myofibers with rimmed vacuoles. No necrotic fibers were detected.

The patient did not report any constitutional or neurological symptoms.

Treatment with intravenous immunoglobulin (0.4 g/kg/day for 5 days) was proposed together with non-pharmacological treatment with cyclic physical rehabilitation. The patient experienced discrete but transient efficacy from the treatment and reported, in particular, a subjective improvement of dysphagia and muscle weakness, despite his need for a walking aid. He subsequently received intravenous immunoglobulin (IVIG) cycles approximately once a year to manage these symptoms. This treatment strategy led to a stabilization of his condition. Physical rehabilitation was also a crucial component of his care.

In June 2023, during a follow-up visit, the patient reported the onset of xerophthalmia, subjective xerostomia, and progressive effort dyspnea. Chest auscultation revealed diminished vesicular breath sounds over the mid-lung fields, absent at the bases, accompanied by bilateral crackles.

### Diagnostic assessment

A high-resolution computed tomography (HRCT) was promptly requested, and it revealed the presence of diffuse reticular thickening of the subpleural interstitium extending bilaterally from the apex to the lung bases, particularly in the middle-lower third, with more concentrated areas in the posterior basal segments of both lower lobes, in the lateral segment of the middle lobe, and in the upper segment of the lingula. Global spirometry demonstrated a mild restrictive ventilatory impairment and a moderate reduction in diffusing capacity of the lung for carbon monoxide (DLCO) (forced vital capacity [FVC]: 77%, forced expiratory volume [FEV1]: 81%, single-breath diffusing capacity for carbon monoxide [DLCO-SB]: 41%, and alveolar volume-adjusted diffusing capacity [DLCO-VA]: 72%).

In the suspicion of secondary lung involvement due to connective tissue disease, an extractable nuclear antigen (ENA) blot was performed, which resulted in positive Ro52 antibodies.

This finding, together with the presence of subjective and objective sicca syndrome, raised the suspicion of SjD diagnosis complicated by ILD. Schirmer’s test was performed, revealing hypolacrimia (2 mm in the right eye and 3 mm in the left eye). To complete the diagnosis, a biopsy of the minor salivary glands (grade 2 according to Chisolm and Mason and focus score = 1) was taken. The patient satisfied the 2016 American College of Rheumatology (ACR)/European League Against Rheumatism (EULAR) classification criteria for Sjögren’s Disease (4, 5). A diagnosis of SjD with interstitial lung disease (ILD) was confirmed. Local treatment for sicca syndrome was initiated. No immunosuppressive therapy was initiated; instead, we preferred to closely monitor pulmonary involvement through a multidisciplinary evaluation to better investigate the clinical behavior.

**Figure 1:**
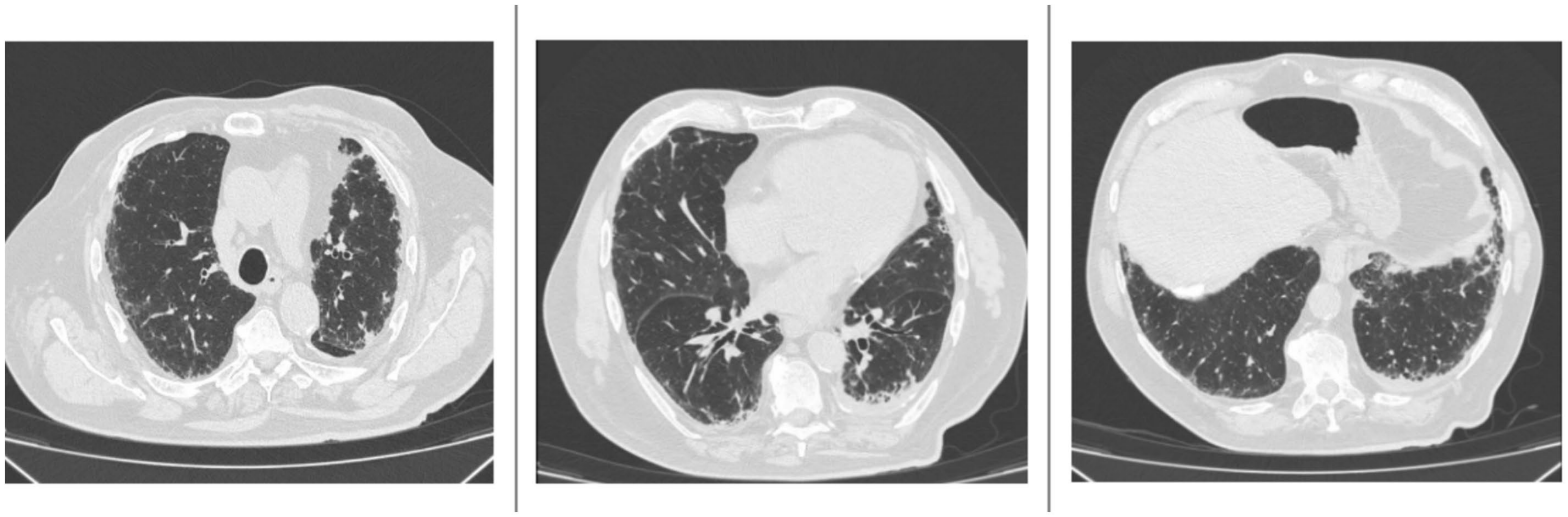
High-resolution computed tomography (HRCT) scans performed in June 2024 show multiple millimetric subpleural bronchiolectases affecting both lower lobes, the lingula, and the middle lobe. There is also a concomitant diffuse fine interstitial reticular thickening, which is also present in the upper lobes.

In April 2024, the patient reported subjective worsening of dyspnea. An HRCT was repeated and revealed progression of the interstitial lung fibrosis with radiologic classification of probable usual interstitial pneumonia (UIP) pattern, characterized by the appearance of multiple millimetric submantellar bronchiolectasis, predominantly in both lower lobes, in the lingular lobe, and in the middle lobe, concomitant with finer interstitial reticular thickening, which was also present in the upper lobes (Figure 1).

A pulmonary function test showed a stable restrictive pattern with stable functional parameters (FVC: 84% and FEV1: 86%). However, the carbon monoxide diffusion test revealed a worsening of values (DLCO-SB: 36% and DLCO-SB 61%). An echocardiography excluded indirect signs of pulmonary hypertension.

The INBUILD criteria for the definition of progressive fibrosing ILD (PF-ILD) were satisfied (5).

### Therapeutic intervention

Following multidisciplinary assessment and the diagnosis of SjD-associated ILD, the patient was treated with combination therapy aimed at controlling both the inflammatory and fibrosing aspects of the disease. Systemic corticosteroid therapy was initiated with prednisone at a dose of 50 mg daily (0.75 mg/kg/day), followed by a slow tapering schedule over 6 months. The tapering regimen involved progressive dose reductions every 2–3 weeks, in order to minimize long-term corticosteroid-related adverse effects. Intravenous cyclophosphamide was administered every month for a period of 6 months according to the National Institutes of Health (NIH) protocol. The patient received a dose of 1.2 g per infusion, calculated based on a body surface area-adjusted range of 0.5–1 g/m^2^. This induction regimen was selected due to its established efficacy in connective tissue disease-associated ILD, particularly in patients with evidence of disease progression or severe inflammatory activity (6). Nintedanib was initially prescribed at the standard dose of 150 mg twice daily, based on current evidence supporting its use in progressive fibrosing ILD (7), including connective tissue disease-associated ILDs.

In accordance with current clinical practice for the management of CTD-ILD and to support further steroid tapering, mycophenolate mofetil at a dose of 2 g/day was prescribed as maintenance therapy.

A few months into therapy, the patient developed gastrointestinal symptoms consistent with dyspepsia, a commonly reported adverse effect of nintedanib.

In response, and prior to clinical consultation, the dosage of nintedanib was reduced to 100 mg bid. This adjustment resulted in partial improvement of symptoms.

Given occult HBV infection (HBsAg− and anti-HBc+), hepatic function was monitored during immunosuppressive therapy according to current guidelines.

### Follow-up and outcomes

The patient reported subjective improvement in swallowing and energy levels following IVIG cycles. He expressed a preference for continued clinical-radiological follow-up rather than invasive diagnostic procedures. Furthermore, after immunosuppressive and antifibrotic therapy, the patient experienced a stabilization of dyspnea.

However, these perceived improvements contrasted with objective findings during follow-up.

**Figure 2:**
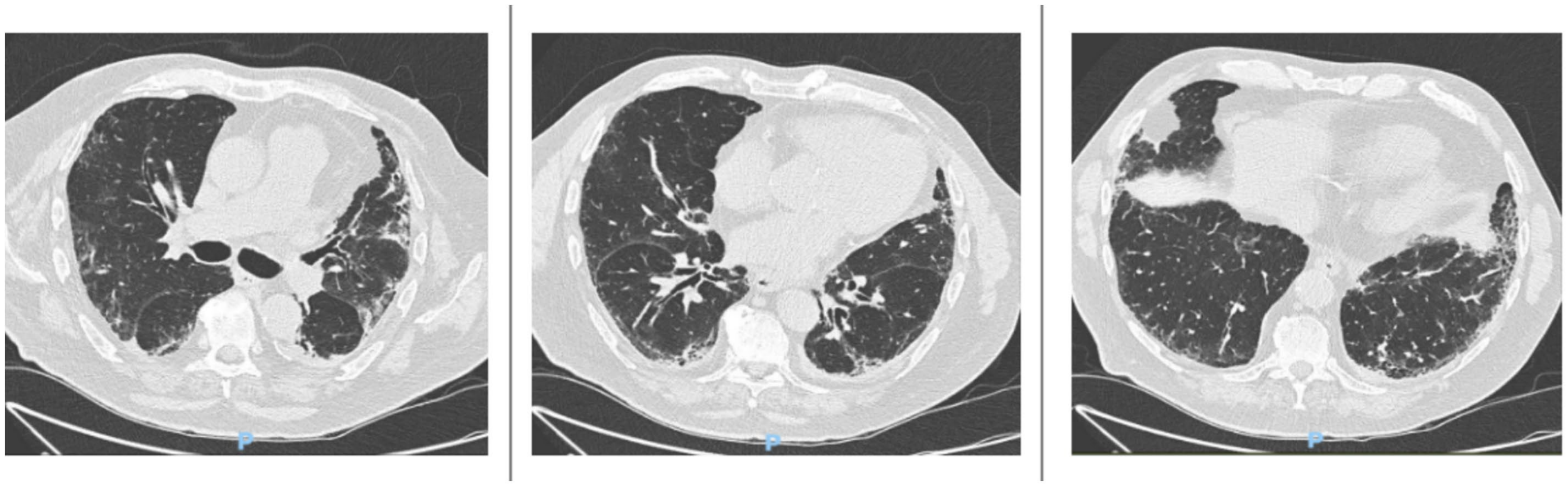
HRCT scans taken in November 2024 reveal a mild progression of interstitial lung disease (ILD). There is a slight increase in traction bronchiolectasis, which is more pronounced in the anterobasal and laterobasal segments of the left lower lobe, the anterior segment of the upper lobe, and the apical segment of the right lower lobe.

In November 2024, an HRCT scan showed mild disease progression, with increased traction bronchiolectasis in the anterobasal and laterobasal segments of the left lower lobe, the anterior segment of the upper lobe, and the apical segment of the right lower lobe (Figure 2).

A 6-min walk test (6MWT) performed during the same period revealed desaturation to 90% on room air after 160 m. The test was limited by the patient’s reduced physical condition.

This apparent discrepancy between the patient’s subjective improvement and the objective evidence of functional and radiological decline highlights the limitations of relying solely on patient-reported outcomes. The patient’s limited mobility prevented an accurate assessment of dyspnea progression, a critical factor for considering therapeutic escalation or for suggesting functional and radiological evaluations beyond routine monitoring and follow-up.

In agreement with the patient, the nintedanib dose was adjusted to 100 mg twice daily to minimize gastrointestinal side effects while maintaining antifibrotic efficacy (8). As of the most recent follow-up, the patient continues combination therapy.

Multidisciplinary follow-up is ongoing.

## Discussion

This case highlights the diagnostic and therapeutic challenges arising from the coexistence of IBM and SjD, particularly when further complicated by ILD.

Although IBM is classified among idiopathic inflammatory myopathies (IIM), it presents with a distinct clinical and histopathological profile. It typically follows an indolent but progressive course, with characteristic involvement of both proximal and distal muscle groups, especially the quadriceps and finger flexors, and is resistant to glucocorticoid therapy.

Dysphagia, as observed in our patient, is a common and often disabling manifestation, resulting from both oropharyngeal and esophageal muscle involvement.

The esophageal dysmotility observed in our patient, confirmed by double-contrast studies, is a hallmark of IBM. Unlike other IIMs, conventional anti-inflammatory and immunosuppressive drugs are often ineffective in IBM; however, intravenous immunoglobulin (IVIG) therapy has demonstrated subjective improvements in muscular weakness and dysphagia in some patients (9). In our case, despite its limited and transient effect, IVIG yielded similar benefits.

SjD is an autoimmune disease primarily affecting exocrine glands such as the salivary and lacrimal glands, typically following an indolent course. However, its systemic involvement is well-recognized, leading to severe manifestations such as ILD, pericarditis, neuropathies, renal insufficiency, and vasculitis. Muscle involvement can also occur, and an association with IIMs has been described. Among patients with both myositis and SjD, IBM is the most common subtype of IIM, with a reported prevalence ranging from 0.5 to 22% (10, 11). Conversely, SjD is also the most frequent connective tissue disease associated with IBM, occurring in 10% of IBM patients (12).

Emerging literature suggests possible shared pathogenic mechanisms between these two diseases, including a common human leukocyte antigen (HLA) background (e.g., HLA A1-B8-DR3-DQ2), the presence of anti-cN1A antibodies (frequently positive in both conditions), and a role for cytotoxic T cells, as supported by the observed association of both diseases with T-cell leukemia and lymphoma (13).

The patient presented with a UIP-like pattern of ILD on high-resolution computed tomography (HRCT), a radiological finding associated with a more aggressive disease course and poorer prognosis in SjD-related ILD (14). Although the literature indicates that UIP-pattern ILD in SjD typically leads to progressive functional decline (15), our patient showed subjective improvement in dyspnea and stabilization of respiratory symptoms following combined antifibrotic and immunosuppressive therapy. However, this symptomatic relief occurred in parallel with mild radiological and functional deterioration, including increased traction bronchiolectasis and decreased exercise tolerance on the 6-min walk test. This apparent contradiction highlights the difficulty in evaluating the patient’s subjective symptoms due to his physical limitations. On the other hand, the mild progression of the interstitial lung disease (ILD) is not in opposition to the treatment’s effectiveness. Given the terrible prognosis of UIP pattern-ILD, the primary goal of therapy is to slow its progression. Therefore, a close follow-up is necessary to accurately study the disease’s course and the patient’s response to treatment.

The coexistence of IBM and ILD also complicates treatment decisions. In our case, the choice of cyclophosphamide for induction therapy was guided by its relatively rapid onset of action and efficacy in progressive connective tissue disease-associated ILD. Although rituximab is also an option, its slower therapeutic onset and less clear benefit for pulmonary involvement influenced our decision. Cyclophosphamide was administered according to the NIH protocol and followed by maintenance therapy with mycophenolate mofetil, which is supported by data from systemic sclerosis-ILD and other CTD-ILDs for maintaining lung function and reducing corticosteroid exposure (16).

Antifibrotic therapy with nintedanib was introduced early due to radiological evidence of PF-ILD. Nintedanib has demonstrated efficacy in slowing FVC decline in patients with progressive fibrosing ILDs, including those associated with autoimmune diseases (17).

Due to gastrointestinal intolerance, the dose was reduced to 100 mg twice daily—a common adjustment in clinical practice that balances tolerability with therapeutic effect.

The patient’s limited mobility precluded an accurate assessment of dyspnea progression, which is a critical factor when considering therapeutic escalation.

For instance, reduced physical capacity due to muscle involvement can hinder the accurate assessment of respiratory function and limit rehabilitation potential. Additionally, dysphagia due to IBM increases the risk of aspiration, which may mimic or exacerbate pulmonary symptoms, potentially confounding ILD monitoring and management.

This case highlights the importance of a multidisciplinary approach involving rheumatologists, pulmonologists, and radiologists in both diagnostic and therapeutic decision-making. Although invasive diagnostic procedures such as bronchoalveolar lavage or lung biopsy could have provided additional diagnostic precision, these were not performed due to patient preference and clinical risk assessment. Nevertheless, longitudinal HRCT imaging and functional assessments allowed us to monitor disease evolution and guide therapy.

Finally, it is important to underline how the combination of immunosuppressive and antifibrotic therapy can change the outcome of these complex patients, leading to the stabilization of the clinical, functional, and radiological picture of interstitial lung involvement. With the emergence of new therapies (such as nerandomilast) (18), we hope to improve the patient’s condition if therapeutic escalation is needed.

## Patient perspective

The patient reported subjective improvement in swallowing and energy levels following IVIG cycles and expressed a preference for continued clinical-radiological follow-up over invasive diagnostic procedures. Furthermore, after immunosuppressive and antifibrotic therapy, the patient experienced a stabilization of dyspnea.

## Data Availability

The raw data supporting the conclusions of this article will be made available by the authors, without undue reservation.
